# Supplementary material for: Evolution and Classification of Musaceae Based on Male Floral Morphology
Source: Plants (Basel). 2023 Apr 10;12(8):1602. doi: 10.3390/plants12081602 (PMC10144554; doi:10.3390/plants12081602)
Supplement: Supplementary file 1 [file plants-12-01602-s001.zip › plants-2277518-supplementary.pdf]

Supporting information

Table S1 List of banana accessions used in the phenetic analysis.

#	Taxon	Accession	Source/Origin
	**Genus:** ***Musa*** **L.**		
	**Section:** ***Musa*** **(included** ***Rhodochlamys*****)**		
1.	*Musa acuminata* Colla subsp. sp.1	SS & JS 104	Chumphon
2.	*Musa acuminata* Colla subsp. sp.1	SS & JS 107	Chumphon
3.	*Musa acuminata* Colla subsp. *malaccensis* (Ridl.) N.W. Simmonds	SS & JS 211	Satun
4.	*Musa acuminata* Colla subsp. *siamea* N.W. Simmonds	SS & JS 001	Phetchabun
5.	*Musa acuminata* Colla subsp. *siamea* N.W. Simmonds	SS & JS 247	Kanchanaburi
6.	*Musa acuminata* Colla subsp. *siamea* N.W. Simmonds	SS & JS 300	Nakon Nayok
7.	*Musa acuminata* Colla subsp. *truncata* (Ridl.) Kiew	SS & JS 206	Yala
8.	*Musa acuminata* Colla subsp. *truncata* (Ridl.) Kiew	SS & JS 493	Narathiwat
9.	*Musa acuminata* Colla subsp. *zebrina* (Van Houtte ex Planch.) Nasution	SS & JS 155	Chiang Mai[1]
10.	*Musa balbisiana* Colla	SS & JS 003	Sukhothai
11.	*Musa balbisiana* Colla	SS & JS 012	Nan
12.	*Musa balbisiana* Colla	SS & JS 354	Mae Hong Son
13.	*Musa flaviflora* (King ex Baker) A.M.Cowan & Cowan	Simmonds 241	India[6, K]
14.	*Musa itinerans* Cheesman	SS & JS 100	Phetchabun
15.	*Musa itinerans* Cheesman	SS & JS 306	Kamphaeng Phet
16.	*Musa itinerans* Cheesman	SS & JS 517	Chiang Mai
17.	*Musa nagensium* Prain	SS & JS 610	Yunnan, China[6, XTBG]
18.	*Musa nanensis* Swangpol & Traiperm	SS & JS 505	Nan
19.	*Musa nanensis* Swangpol & Traiperm	SS & JS 527	Nan
20.	*Musa ornata Roxb.* 'Bua Si Muang'	SS & JS 169	Nakhon Ratchasima[4]
21.	*Musa ornata* Roxb. 'Bua Luang'	SS & JS 433	Kamphaeng Phet[4]

#	Taxon	Accession	Source/Origin
22.	*Musa ornata* Roxb. 'Bua Si Chomphu'	SS & JS 515	Bangkok[4]
23.	*Musa ornata* Roxb. 'Bua Si Chomphu '	SS & JS 542	Chiang Mai[3]
24.	*Musa ornata* Roxb. 'Bua Si Muang'	SS & JS 543	Chiang Mai[3]
25.	*Musa ornata* Roxb. 'Bua Luang'	SS & JS 568	Bangkok[4]
26.	*Musa ornata Roxb.* 'Bua Khao'	SS & JS 593	Chiang Mai[3]
27.	*Musa rubra* Wall. ex Kurz	SS & JS 352	Mae Hong Son
28.	*Musa rubra* Wall. ex Kurz	SS & JS 420	Mae Hong Son
29.	*Musa rubra* Wall. ex Kurz	SS & JS 516	Bangkok[4]
30.	*Musa serpentina* Swangpol & Somana	SS & JS 246	Kanchanaburi
31.	*Musa serpentina* Swangpol & Somana	SS & JS 353	Mae Hong Son
32.	*Musa serpentina* Swangpol & Somana	SS & JS 355	Mae Hong Son
33.	*Musa siamensis* Häkkinen & Rich.H.Wallace	SS & JS 524	Bangkok[4]
34.	*Musa velutina* H.Wendl. & Drude	SS & JS 520	Chiang Mai[1]
35.	*Musa* cf. *yunnanensis*	SS & JS 255	Chiang Mai
36.	*Musa* cf. *yunnanensis*	SS & JS 444	Chiang Mai
37.	*Musa* cf. *yunnanensis*	SS & JS 531	Chiang Mai
	**Section : *Callimusa* (included *Australimusa*)**		
38.	*Musa beccarii* N.W.Simmonds	SS & JS 539	Bangkok[4]
39.	*Musa coccinea* Andrews	SS & JS 223	Phetchabun[4]
40.	*Musa coccinea* Andrews	SS & JS 519	Chiang Mai[4]
41.	*Musa ingens* N.W.Simmonds	B. E. 40	Papua New Guinea[6, K]
42.	*Musa gracilis* Holttum	SS & JS 487	Narathiwat
43.	*Musa gracilis* Holttum	SS & JS 488	Narathiwat
44.	*Musa haekkinenii* N.S.Lý & Haev.	SS & JS 540	Bangkok[4]
45.	*Musa maclayi* F.Muell. ex Mikl.-Maclay	Carr 15590	Papua New Guinea[6, K]
46.	*Musa maclayi* F.Muell. ex Mikl.-Maclay subsp. maclayi	B.E. 40	Papua New Guinea[6, K]
47.	*Musa maclayi* F.Muell. ex Mikl.-Maclay	Poulsen 3132	Papua New Guinea[6, K]
48.	*Musa paracoccinea* A.Z.Liu & D.Z.Li	SS & JS 611	Yunnan, China[5]

#	Taxon	Accession	Source/Origin
49.	*Musa textilis* Née	P.F. Stevens et al 461	Sabah, Malesia[6, E]
	**Genus: *Ensete* Horan.**		
50.	*Ensete glaucum* (Roxb.) Cheesman	SS & JS 229	Bangkok[4]
51.	*Ensete glaucum* (Roxb.) Cheesman	SS & JS 244	Kanchanaburi
52.	*Ensete glaucum* (Roxb.) Cheesman	SS & JS 317	Tak
53.	*Ensete gilletii* (= *livingstonianum*) (De Wild.) Cheesman	Keay 28003	Nigeria[6, K]
54.	*Ensete homblei* (Bequaert ex De Wild.) Cheesman	Milne-Redhead 3062	Zimbabwe, Rhodesia[6, K]
55.	*Ensete superbum* (Roxb.) Cheesman	SS & JS 242	Kanchanaburi
56.	*Ensete superbum* (Roxb.) Cheesman	SS & JS 391	Kanchanaburi
57.	*Ensete superbum* (Roxb.) Cheesman	SS & JS 541	Chiang Mai[1]
	**Genus: *Musella* C.Y. Wu**		
58.	*Musella lasiocarpa* (Franch.) C.Y. Wu ex H.W. Li		
		SS & JS 231	Nakhon Ratchasima[4]
59.	*Musella lasiocarpa* (Franch.) C.Y. Wu ex H.W. Li	SS & JS 594	Phetburi collection[4]

[1] QSBG (Queen Sirikit Botanic Garden, Chiang Mai, Thailand); [2] banana collection, Bangkok, Thailand; [3] Royal Project Inthanon, Chiang Mai, Thailand; [4] Cultivated bananas in Thailand; [5] Cultivated bananas in different countries; [6] Wild bananas collected in different countries deposited in the herbaria: E = RBG Edinburgh; K = RBG Kew; XTBG = Xishuangbanna Tropical Botanical Garden

Table S2. List of sequences of Zingiberales-Musaceae used in the phylogenetic analysis.

No.	Family	Species	Accession	ITS	*rps16*	*trnL-F*	*atpB-rbcL*
1.	Typhaceae	*Sparganium* sp.	-	-	HQ913892	HQ882765	JF280745
2.		*Typha angustifolia*	-	-	AM116858	JF319450	FJ914237
3.	Cannaceae	*Canna indica*	19981130-03 (BR)	KU215028	KU214891	KU215151	-
4.		*Canna paniculata*	-	AY673069	AY656159	AY140423	-
5.	Costaceae	*Chamaecostus cuspidatus*	19381338 (BR)	KU215041	-	KU215165	-
6.		*Chamaecostus subsessilis*	-	AY994717	KJ011420	AY994555	-
7.		*Costus allenii*	19730144 (BR)	KU215031	KU214894	KU215154	KU215248
8.		*Costus amazonicus*	-	AY041032	KJ011354	AY994586	-
9.		*Costus arabicus*	19730142 (BR)	KU215034	KU214897	KU215156	KU215251
10.		*Costus barbatus*	-	AY041031	KJ011357	AY994585	-
11.		*Costus chartaceus*	-	AY972911	KJ011360	AY994559	-
12.		*Costus claviger*	-	AY972882	KJ011361	AY994584	-
13.		*Costus deisetii*	19615174 (BR)	KU215038	KU214901	-	KU215255
14.		*Costus dinklagei*	-	AY994750	KJ011366	AY994596	-
15.		*Costus dubius*	19594350 (BR)	KU215040	KU214903	KU215161	KU215257
16.		*Costus erythrocoryne*	-	AY972886	-	AY994579	-
17.		*Costus erythrophyllus*	19730145 (BR)	KU215030	KU214893	KU215153	KU215247
18.		*Costus gabonensis*	-	AY994747	KJ011371	AY994593	-
19.		*Costus guanaiensis*	-	AY972883	KJ011374	AY994577	-
20.		*Costus laevis*	-	AY041035	KJ011377	AY994575	-
21.		*Costus lateriflorus*	-	AY994734	KJ011379	AY994574	-
22.		*Costus letestui*	-	AY972939	KJ011380	AY994573	-
23.		*Costus lucanusianus*	19620189 (BR)	KU215039	KU214902	KU215160	KU215256
24.		*Costus maculatus*	-	AY994731	-	AY994571	-
25.		*Costus malortienanus*	10005061 (BR)	KU215029	KU214892	KU215152	KU215246
26.		*Costus montanus*	-	KU215032	KU214895	-	KU215249
27.		*Costus mosaicus*	-	AY994728	-	AY994568	-
28.		*Costus phaeotrichus*	-	AY994721	KJ011396	AY994561	-
29.		*Costus pictus*	10005272 (BR)	KU215033	KU214896	KU215155	KU215250
30.		*Costus plicatus*	-	AY041030	KJ011400	AY994565	-

No.	Family	Species	Accession	ITS	*rps16*	*trnL-F*	*atpB-rbcL*
31.		*Costus pulverulentus*	-	AY041029	AY656160	AY994563	-
32.		*Costus scaber*	19812689 (BR)	KU215035	KU214898	KU215157	KU215252
33.		*Costus spectabilis*	-	AY994718	KJ011406	AY994556	-
34.		*Costus stenophyllus*	-	AY994720	KJ011408	AY994560	-
35.		*Costus talbotii*	-	AY994716	KJ011412	AY994554	-
36.		*Costus tappenbeckianus*	-	AY994715	-	AY994553	-
37.		*Costus vargasii*	19860011 (BR)	KU215037	KU214900	KU215159	KU215254
38.		*Costus varzearum*	-	AY994714	KJ011413	AY994551	-
39.		*Costus villosissimus*	-	AY994713	KJ011414	AY994550	-
40.		*Costus zingiberoides*	19860010 (BR)	KU215036	KU214899	KU215158	KU215253
41.		*Dimerocostus strubilaceus*	19726435 (BR)	-	KU214904	KU215162	KU215258
42.		*Monocostus uniflora*	19750179 (BR)	-	KU214905	KU215163	KU215259
43.		*Tapeinochilos ananasae*	19610260 (BR)	-	KU214906	KU215164	KU215260
44.	Heliconiaceae	*Heliconia acuminata*	19830761 (BR)	-	-	KU215166	KU215261
45.		*Heliconia angusta*	19750486 (BR)	-	-	KU214909	-
46.		*Heliconia caribaea*	-	FJ428106	FJ428109	FJ428179	FJ428018
47.		*Heliconia densiflora*	19880067 (BR)	-	-	-	KU215263
48.		*Heliconia hirsuta*	Van Caeckenberghe s.n. (BR)	-	-	KU214912	KU215267
49.		*Heliconia humilis*	19861386 (BR)	-	KU214910	KU215168	KU215265
50.		*Heliconia illustris*	10005273 (BR)	-	KU214907	-	KU215262
51.		*Heliconia indica*	19730253 (BR)	-	KU214908	KU215167	KU215264
52.		*Heliconia psittacorum*	-	FJ428105	FJ428108	FJ428180	FJ428016
53.		*Heliconia rickardiana*	19940052-53 (BR)	-	KU214913	-	KU215268
54.		*Heliconia rostrata*	19822412 (BR)	KU215042	KU214907	KU215169	KU215266
55.		*Orchidantha chinensis*	-	FJ428181	FJ428153	FJ428181	FJ428061
56.		*Orchidantha fimbriata*	-	AF434879	AF430098	FJ621300	-
57.		*Orchidantha maxillarioides*	19074031 (BR)	-	KU214914	KU215170	KU215269
58.		*Orchidantha siamensis*	-	AF434887	AF430106	AF431622	-
59.	Marantaceae	*Afrocalathea rhizantha*	-	EU605908	EF382847	EU647816	-
60.		*Calathea altissima*	-	JQ341268	AF141025	JN413119	-
61.		*Calathea capitata*	-	JQ341271	AF141026	JQ341219	-

No.	Family	Species	Accession	ITS	*rps16*	*trnL-F*	*atpB-rbcL*
62.		*Calathea mackeyana*	19073768 (BR)	KU215049	KU214921	KU215175	KU215274
63.		*Calathea majestica*	19700532 (BR)	KU215051	KU214923	KU215177	KU215276
64.		*Calathea marantifolia*	19760485 (BR)	KU215043	KU214915	KU215171	KU215270
65.		*Calathea metallica*	-	AY673046	AF141030	AY140354	-
66.		*Calathea micans*	-	JQ341289	AF141031	JN413140	-
67.		*Calathea microcephala*	-	-	KU214932	-	KU215281
68.		*Calathea mirabilis*	-	KU215045	KU214917	KU215173	-
69.		*Calathea petersenii*	-	JQ341294	AF141032	JQ341237	-
70.		*Calathea picturata*	Van Caeckenberghe s.n. (BR)	KU215055	KU214928	-	-
71.		*Calathea rufibarba*	-	AY673048	AF141035	AY140360	-
72.		*Calathea undulata*	Van Caeckenberghe s.n. (BR)	KU215273	KU215048	KU214920	-
73.		*Calathea variegata*	19820964 (BR)	-	KU215047	KU214919	KU215272
74.		*Calathea warscewiczii*	-	AY673049	AY656139	AY140364	-
75.		*Calathea zebrina*	19700743 (BR)	KU215053	KU214925	-	-
76.		*Ctenantha amabilis*	19391944 (BR)	KU215058	KU214930	KU215178	KU215279
77.		*Ctenantha kummeriana*	19073861 (BR)	KU215056	-	-	KU215277
78.		*Ctenantha oppenheimiana*	19680371 (BR)	KU215059	KU214931	KU215179	KU215280
79.		*Ctenantha setosa*	19620507 (BR)	KU215057	KU214929	KU215190	KU215278
80.		*Donax canniformis*	19733425 (BR)	KU215069	KU214945	KU215191	-
81.		*Groeppertia louisea*	Van Caeckenberghe s.n. (BR)	KU215046	KU214918	KU215174	KU215271
82.		*Halopegia azurea*	-	AY914650	AF141048	AY140372	-
83.		*Hypselodephys hirsuta*	Hypselodelphys hirsuta	KU215064	KU214939	KU215186	KU215288
84.		*Indianthus virgatus*	-	AY914666	AY914620	AY140411	-
85.		*Ischnosiphon heleniae*	-	AY673055	AY656145	AY140379	-
86.		*Ischnosiphon leucophaeus*	-	JQ341309	AF141053	JN413162	-
87.		*Ischnosiphon ovatus*	Van Caeckenberghe s.n. (BR)	KU215067	KU214942	-	KU215291
88.		*Maranta bicolor*	19074039 (BR)	KU215061	KU214934	KU215181	KU215283
89.		*Maranta depressa*	19700747 (BR)	KU215060	KU214933	KU215180	KU215282
90.		*Maranta leuconeura*	19660030 (BR)	KU215062	KU214935	KU215182	KU215284
91.		*Marantochloa conferta*	Van Caeckenberghe s.n. (BR)	-	KU214943	KU215188	-
92.		*Marantochloa congensis*	-	EU605903	AF141062	EU647811	-

No.	Family	Species	Accession	ITS	*rps16*	*trnL-F*	*atpB-rbcL*
93.		*Marantochloa filipes*	19850681 (BR)	KU215070	KU214946	KU215192	-
94.		*Marantochloa leucantha*	19547056 (BR)	-	KU214938	KU215185	KU215287
95.		*Marantochloa purpurea*	-	AY673057	AF141067	AY140389	-
96.		*Megaphrynium macrostachyum*	10005583 (BR)	KU215071	KU214947	KU215193	-
97.		*Monotagma laxum*	-	AY673058	AY656148	AY140392	-
98.		*Phrynium giganteum*	-	AY673050	EF382848	AY140365	-
99.		*Phrynium imbricatum*	-	AY673059	AY656149	AY140402	-
100.		*Phrynium maximum*	-	AF434901	AF430118	AY140398	-
101.		*Phrynium pubinerve*	-	JQ341264	AY914638	JQ341212	-
102.		*Pleiostachya pruinosa*	19910154-31 (BR)	KU215063	KU214937	KU215184	KU215286
103.		*Sarantha* sp.	19575002 (BR)	KU215065	KU214940	KU215187	KU215289
104.		*Sarcophrynium brachystachyum*	19910155-32 (BR)	KU215072	KU214948	KU215194	KU215285
105.		*Sarcophrynium priogonium*	10005466 (BR)	-	KU214936	KU215183	-
106.		*Schumannianthus dichotomus*	-	AY673064	AY656154	AY140410	-
107.		*Stachyphrynium latifolium*	-	AY914653	AY914607	AY140412	-
108.		*Stachyphrynium repens*	-	AY673060	AY656150	AY140403	-
109.		*Stachyphrynium spicatum*	-	AY914658	AY914612	AY140415	-
110.		*Stachyphrynium sumatranum*	-	AY914659	AY914613	AY140400	-
111.		*Stromanthe partema*	19710269 (BR)	KU215066	KU214941	-	KU215290
112.		*Thalia dealbata*	-	AY914693	AY914648	AY140419	-
113.		*Thalia multiflora*	19770092 (BR)	KU215068	KU214944	KU215189	KU215292
114.		*Thaumatococcus daniellii*	-	AY673067	AF141091	AY140421	-
115.		*Trachyphrynium braunianum*	-	EU605916	AY656158	AY140377	-
116.	Musaceae	*Ensete gilletii*	ITC1389	KU215101	KU214977	-	KU215319
117.		*Ensete glaucum*	-	FJ428154	FJ428124	FJ428154	FJ428019
118.		*Ensete glaucum*	ITC0775	KU215088	KU214963	-	KU215306
119.		*Ensete homblei*	-	FJ621290	-	FJ621290	-
120.		*Ensete superbum*	-	FJ621291	-	FJ621291	-
121.		*Ensete superbum*	SS & JS 583	ON639613	ON453859	ON453869	ON453850
122.		*Ensete ventricosum*	ITC1387	KU215100	KU214976	KU215215	-
123.		*Musa acuminata* subsp. *banksii*	ITC0617	KU215083	KU214959	KU215206	KU215302

No.	Family	Species	Accession	ITS	*rps16*	*trnL-F*	*atpB-rbcL*
124.		*Musa acuminata* subsp. *burmannica*	ITC0283	FJ428083	FJ428135	FJ428169	FJ428041
125.		*Musa acuminata* subsp. *burmannicoides*	ITC0249	FJ428085	FJ428133	FJ428170	FJ428044
126.		*Musa acuminata* subsp. sp.1	SS & JS 600	ON639614	ON453860	ON453870	ON453860
127.		*Musa acuminata* subsp. *malaccensis*	ITC0609	KU176107	KU176108	KU176109	-
128.		*Musa acuminata* subsp. *malaccensis*	ITC1511	KU215102	KU214978	KU215205	KU215320
129.		*Musa acuminata* subsp. *microcarpa*	ITC0308	KU215076	KU214952	KU215198	KU215296
130.		*Musa acuminata* subsp. *microcarpa*	ITC0253	FJ428087	FJ428140	FJ428174	FJ428052
131.		*Musa acuminata* subsp. *siamea*	ITC0672	KU215122	KU214997	-	KU215338
132.		*Musa acuminata* subsp. *siamea*	SS & JS 173	ON639615	ON453861	KT257581	ON453852
133.		*Musa acuminata* subsp. *truncata*	ITC0393	KU215124	KU214999	KU215218	KU215340
134.		*Musa acuminata* subsp. *zebrina*	ITC0728	KU215087	KU214962	-	-
135.		*Musa acuminata* subsp. *zebrina*	ITC1177	KU215097	KU214973	-	KU215316
136.		*Musa acuminata* subsp. *zebrina*	ITC1178	KU215098	KU214974	-	KU215317
137.		*Musa acuminata* subsp. *zebrina*	ITC1179	KU215099	KU214975	-	KU215318
138.		*Musa aurantiaca*	HBG: 2007–0001	FJ428090	FJ428127	FJ428162	FJ428037
139.		*Musa balbisiana*	ITC0247	KU215074	KU214950	KU215196	KU215294
140.		*Musa balbisiana*	ITC0565	KU215080	KU214956	KU215202	KU215300
141.		*Musa balbisiana*	ITC1587	KU215114	KU214989	-	KU215330
142.		*Musa barioensis*	ITC1568	KU215112	KU214987	-	KU215328
143.		*Musa basjoo*	ITC0061	KU215073	KU214949	KU215195	KU215293
144.		*Musa beccarii* var. *beccarii*	HBG: 2001–0482	FJ428065	FJ428120	FJ428189	FJ428028
145.		*Musa beccarii* var. *hottana*	HBG: 2005–0826	FJ428066	FJ428115	FJ428190	FJ428029
146.		*Musa borneensis*	ITC1531	KU215110	KU214985	-	KU215326
147.		*Musa campestris* subsp. *sarawakensis*	ITC1517	KU215104	KU214980	-	KU215322
148.		*Musa cheesmanii*	ITC1519	KU215106	KU214982	-	KU215323
149.		*Musa coccinea*	ITC0287	KU215078	KU214954	KU215200	KU215298
150.		*Musa exotica*	ITC1532	KU215111	KU214986	-	KU215327
151.		*Musa gracilis*	HBG: 2001–0452	FJ428075	FJ428111	FJ428194	FJ428022
152.		*Musa hirta*	HBG: 2004–0366	FJ428074	FJ428117	FJ428199	FJ428026
153.		*Musa ingens*	HBG: 2005–0375	FJ428077	FJ428118	FJ428184	FJ428036
154.		*Musa itinerans* subsp. *xishuangbanaensis*	ITC1526	KU215108	KU214984		KU215325

No.	Family	Species	Accession	ITS	*rps16*	*trnL-F*	*atpB-rbcL*
155.		*Musa jackeyi*	ITC0588	KU215081	KU214957	KU215203	KU215301
156.		*Musa laterita*	HBG: 2001–0448	FJ428082	FJ428136	FJ428157	FJ428033
157.		*Musa lolodensis*	ITC0956	KU215094	KU214970	KU215213	KU215313
158.		*Musa lutea*	ITC1515	KU215103	KU214979	-	KU215321
159.		*Musa maclayi*	ITC0864	FJ428068	FJ428122	FJ428183	FJ428032
160.		*Musa maclayi* subsp. *ailuluai*	ITC0614	KU215116	KU214991	KU215216	KU215332
161.		*Musa maclayi* subsp. *maclayi*	ITC0934	KU215119	KU214994	-	KU215335
162.		*Musa maclayi* subsp. *maclayi* var. *maclayi*	ITC0915	KU215091	KU214967	KU215212	KU215310
163.		*Musa mannii*	ITC0543	KU215079	KU214955	KU215201	KU215299
164.		*Musa monticola*	HBG: 2004–0365	FJ428073	FJ428119	FJ428191	FJ428049
165.		*Musa nagensium*	HBG: 2006–0700	FJ428101	FJ428144	FJ428158	FJ428058
166.		*Musa nanensis*	SS & JS 612	ON639616	ON453853	ON453871	ON453862
167.		*Musa ornata*	ITC0637	KU215117	KU214992	-	KU215333
168.		*Musa ornata*	SS & JS 169	ON639617	ON453863	KT257590	ON453854
169.		*Musa peekelii* subsp. *angustigemma*	ITC0618	KU215084	KU214960	KU215207	KU215303
170.		*Musa peekelii* subsp. *angustigemma*	ITC0625	KU215086	KU214961	KU215209	KU215305
171.		*Musa peekelii* subsp. *peekelii*	ITC0917	KU215092	KU214968	-	KU215311
172.		*Musa rosea*	HBG: 2001–0401	FJ428080	FJ428131	FJ428171	FJ428045
173.		*Musa rubinea*	HBG: 2003–0768	FJ428093	FJ428128	FJ428163	FJ428048
174.		*Musa rubra*	ITC1590	KU215115	KU214990	-	KU215331
175.		*Musa rubra*	HBG: 2001–0402	FJ428081	FJ428132	FJ428172	FJ428046
176.		*Musa salaccensis*	HBG: 2003–0784	FJ428072	FJ428112	FJ428196	FJ428023
177.		*Musa schizocarpa*	ITC0599	KU215082	KU214958	KU215204	-
178.		*Musa schizocarpa*	ITC0890	-	KU214965	KU215210	KU215308
179.		*Musa schizocarpa*	ITC0926	KU215093	KU214969	-	KU215312
180.		*Musa schizocarpa*	ITC1002	KU215120	KU214995	-	KU215336
181.		*Musa serpentina*	SS & JS 353	ON639618	ON453864	KT257594	ON453855
182.		*Musa siamensis*	HBG: 2002–0844	FJ428086	FJ428134	FJ428168	FJ428047
183.		*Musa textilis*	ITC0539	KU215095	KU214971	KU215214	KU215314
184.		*Musa tonkinensis*	HBG: 2001–0392	FJ428099	FJ428146	FJ428178	FJ428055
185.		*Musa velutina*	ITC0638	KU215118	KU214993	-	KU215334

No.	Family	Species	Accession	ITS	*rps16*	*trnL-F*	*atpB-rbcL*
186.		*Musa violascens*	ITC1514	KU215123	KU214998	KU215217	KU215339
187.		*Musa yunnanensis*	ITC1573	KU215113	KU214988	-	KU215329
188.		*Musa* cf. *yunnanensis*	SS & JS 444	KT257625	ON453865	KT257599	-
189.		*Musella lasiocarpa*	HBG: 2005–0824	FJ428155	FJ428123	FJ428155	FJ428021
190.	Strelitziaceae	*Pherakospermum guianese*	19812682 (BR)		KU215000	KU215219	
191.		*Ravenala madagascariensis*	SCBG: 2005013	FJ428107	FJ428110	FJ428182	FJ428017
192.		*Strelitzia reginae*	-	FJ626403	JQ027166	FJ621298	
193.	Zingiberaceae	*Aframomum danielii*	20030092-77 (BR)	KU215148	KU215024	KU215239	KU215362
194.		*Aframomum hanburyi*	Van Caeckenberghe s.n. (BR)	KU215147	KU215023		KU215361
195.		*Aframomum korima*	Van Caeckenberghe s.n. (BR)	KU215150		KU215241	KU215364
196.		*Aframomum luteoalbum*	-	AF414493	AF414546	FJ848664	
197.		*Aframomum thoneri*	20030090-75 (BR)	KU215149		KU215240	KU215363
198.		*Aframomum verrucosum*	-	AF414492	AF414545	FJ848660	
199.		*Alpinia luteocarpa*	19880195 (BR)	KU215125	KU215001	KU215220	KU215342
200.		*Alpinia pupurata*	19880109 (BR)	KU215126	KU215002	KU215221	KU215343
201.		*Boesenbergia pandurata*	20040188-85 (BR)		KU215139	KU215016	
202.		*Boesenbergia* sp.	19710472 (BR)	KU215135	KU215012	KU215229	
203.		*Cautleya lutea*	20091405-86 (BR)	KU215141	KU215018	KU215234	KU215355
204.		*Curcuma longa*	19670244 (BR)	-	KU215006	KU215224	KU215347
205.		*Curcuma zanthorrhiza*	19750147 (BR)	KU215130	KU215007	-	KU215348
206.		*Etlingera elatior*	19560132 (BR)	-	KU215025	KU215242	-
207.		*Etlingera yunnanensis*	-	AF414468	AF414521	AY769809	-
208.		*Globba schlombergii*	19741200 (BR)	KU215143	KU215020	KU215236	KU215357
209.		*Hedychium corosarium*	19870230 (BR)	KU215134	KU215011	KU215228	-
210.		*Hedychium cylindrica*	19520915 (BR)	KU215132	KU215009	KU215226	KU215350
211.		*Kaempferia elegans*	20060023-35 (BR)	KU215138	KU215015	KU215232	KU215353
212.		*Hedychium flavescens*	19800483 (BR)	KU215133	KU215010	KU215227	KU215351
213.		*Hedychium horsfieldii*	19602071 (BR)	KU215131	KU215008	KU215225	KU215349
214.		*Kaempferia gibbertii*	19570275 (BR)	KU215136	KU215013	KU215230	KU215352
215.		*Kaempferia rotunda*	19074013 (BR)	KU215137	KU215014	KU215231	-
216.		*Kaempferia* sp.	Van Caeckenberghe s.n. (BR)	KU215146	KU215022	KU215238	KU215360

No.	Family	Species	Accession	ITS	*rps16*	*trnL-F*	*atpB-rbcL*
217.		*Reanalmia alpina*	19921107-23 (BR)	KU215144	KU215021	-	KU215358
218.		*Reanalmia nicolaioides*	19750423 (BR)	KU215140	KU215017	KU215233	KU215354
219.		*Renealmia cernua*	-	AF414476	AF414529	DQ444517	-
220.		*Riedelia* sp.	19620382 (BR)	KU215145	-	KU215237	KU215359
221.		*Siphonochilus decorus*	19870078 (BR)	-	KU215026	KU215243	-
222.		*Siphonochilus kirkii*	20040213-13 (BR)	KU215142	KU215019	KU215235	KU215356
223.		*Zingiber darceyi*	Van Caeckenberghe s.n. (BR)	KU215129	KU215005	-	KU215346
224.		*Zingiber officinale*	19920009-89 (BR)	-	KU215027	KU215244	-
225.		*Zingiber papuarua*	19763771 (BR)	KU215127	KU215003	KU215222	KU215344
226.		*Zingiber zerumber*	19520932 (BR)	KU215128	KU215004	KU215223	KU215345

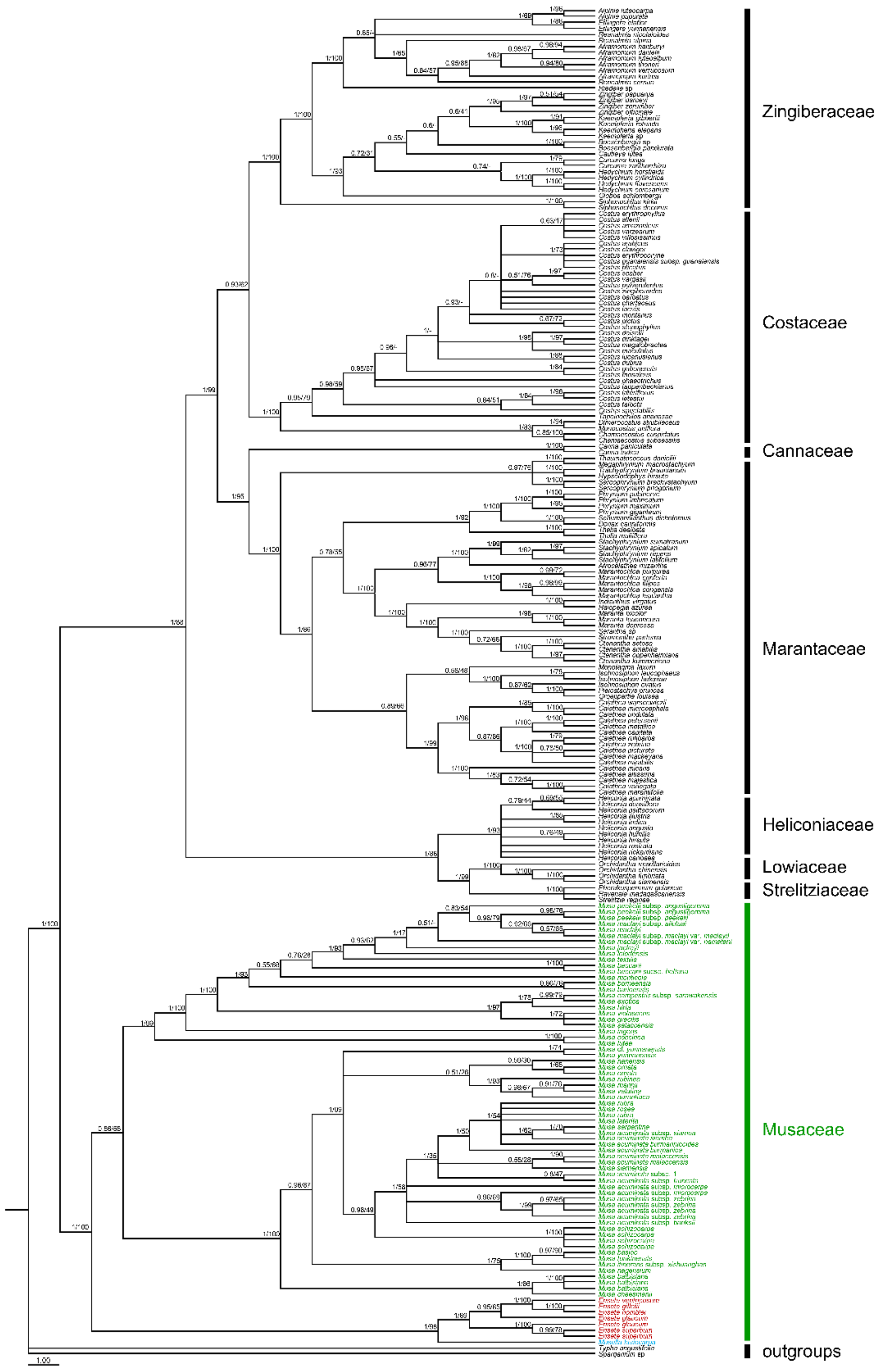

Figure S1. Bayesian 50% Majority role consensus cladogram of the combined ITS, *rps16*, *trnL-F* and *atpB-rbcL* dataset. The tree separated three genera including *Musa* (green), *Ensete* (red) and *Musella* (blue). Bayesian posterior probabilities (PP) and bootstrap supports are labelled on the nodes.

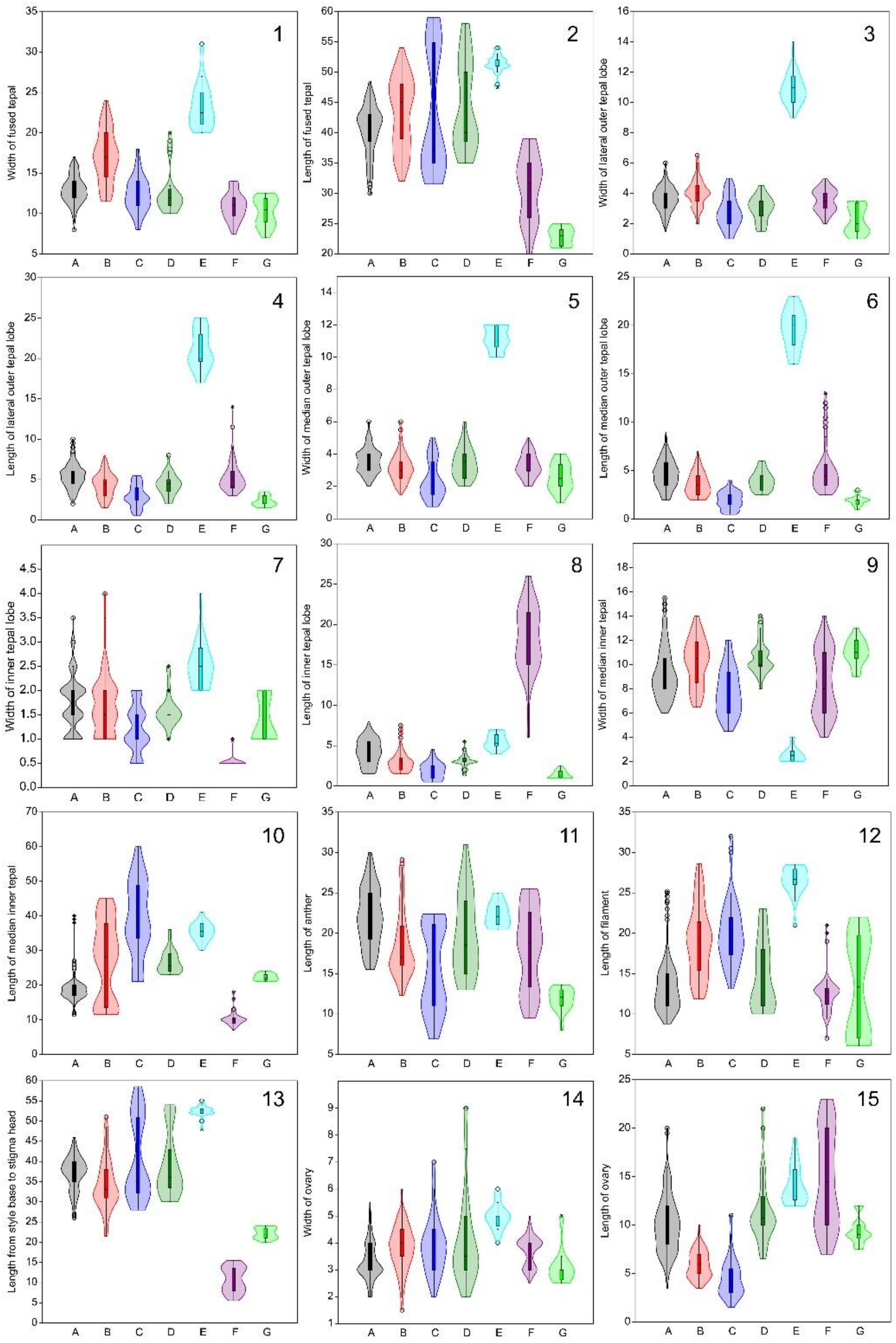

Figure S2(1-15) Distributed data of 15 measurements of male floral organs, (1) width of fused tepal, (2) length of fused tepal, (3) width of lateral outer tepal lobe, 4) length of lateral outer tepal lobe, (5) width of median outer tepal lobe, (6) length of median outer tepal lobe, (7) width of inner tepal lobe, (8) length of inner tepal lobe, (9) width of median inner tepal, (10) length of median inner tepal, (11) length of anther, (12) length of filament, (13) length from style base to stigma head, (14) width of ovary and (15) length of ovary. Dots and stars indicate outlying values. The banana groups are labelled as follows: (A) the section *Musa*, (B) the section *Rhodochlamys*, (C) the section *Callimusa*, (D), the section *Australimusa*, (E) *Musa nanensis*, (F) *Ensete* and (G) *Musella*.
